# Taxonomic composition of the particle-attached and free-living bacterial assemblages in the Northwest Mediterranean Sea analyzed by pyrosequencing of the 16S rRNA

**DOI:** 10.1002/mbo3.92

**Published:** 2013-05-31

**Authors:** Bibiana G Crespo, Thomas Pommier, Beatriz Fernández-Gómez, Carlos Pedrós-Alió

**Affiliations:** 1 Departament de Biologia Marina i Oceanografia Institut de Ciències del Mar, CSIC08003, Barcelona, Spain; 2 Ecologie Microbienne UMR5557, USC1193 CNRS, INRA, Université Lyon I69622, Villeurbanne, France

**Keywords:** Marine bacteria, microbial communities, microbial ecology, particle attached, pyrosequencing

## Abstract

Free-living (FL) and particle-attached (PA) bacterial assemblages in the Northwest Mediterranean Sea were studied using pyrosequencing data of the 16S rRNA. We have described and compared the richness, the distribution of Operational Taxonomic Units (OTUs) within the two fractions, the spatial distribution, and the taxonomic composition of FL and PA bacterial assemblages. The number of OTUs in the present work was two orders of magnitude higher than in previous studies. Only 25% of the total OTUs were common to both fractions, whereas 49% OTUs were exclusive to the PA fraction and 26% to the FL fraction. The OTUs exclusively present in PA or FL assemblages were very low in abundance (6% of total abundance). Detection of the rare OTUs revealed the larger richness of PA bacteria that was hidden in previous studies. Alpha-*Proteobacteria* dominated the FL bacterial assemblage and gamma-*Proteobacteria* dominated the PA fraction. *Bacteroidetes* were important in the PA fraction mainly at the coast. The high number of sequences in this study detected additional phyla from the PA fraction, such as *Actinobacteria, Firmicutes,* and *Verrucomicrobia*.

## Introduction

Heterotrophic bacteria play fundamental roles in marine biogeochemical cycles mediating organic matter mineralization. A significant fraction of the degradation of organic matter takes place on particles, where carbon and nutrient concentrations are much higher than in the surrounding waters (Cammen and Walker 1982). The outcome of this decomposition is relevant for the amount of primary production that either sinks to the bottom or is recycled in the photic layer. The degradation of organic matter on particles occurs by the activity of bacteria with specific adaptations. The particle-attached bacteria (PA bacteria) are bigger and are present in higher local concentrations than those found free living (FL bacteria) in the water (Caron et al. 1982; Pedrós-Alió and Brock 1983; Fernández-Gómez et al. 2013). PA bacteria have also been shown to incorporate more substrates and to present higher hydrolysis rates than the FL bacteria (Pedrós-Alió and Brock 1983; Simon et al. 2002). PA bacteria need specific adaptations to be able to attach to the surface of particles and degrade the organic matter compounds (Bauer et al. 2006). Thus, the PA assemblage may be expected to be different from the FL assemblage. Moreover, different attached bacteria have different hydrolytic enzymatic activities (Karner and Herndl 1992; Martínez et al. 1996). Therefore, changes in organic matter composition in particles may cause changes in the species composition of the PA bacterial assemblages (Karner and Herndl 1992; Pinhassi et al. 1999).

In addition, PA bacteria must be capable of surviving freely in the water column so they can colonize new particles (Pedrós-Alió and Brock 1983; Ghiglione et al. 2007) and therefore they will contribute, at particular times, to the FL bacterial community. This fact will influence the degree of similarity between PA and FL bacterial communities.

Only a few studies have analyzed the identity of bacteria in particles (DeLong et al. 1993; Rath et al. 1998; Acinas et al. 1999; Hollibaugh et al. 2000; Ghiglione et al. 2007; Kellogg and Deming 2009). Several of them have shown that PA bacteria are phylogenetically different from FL bacteria, not only in marine systems (DeLong et al. 1993; Acinas et al. 1999) but also in estuaries (Bidle and Fletcher 1995) and in coastal lagoons (LaMontagne and Holden 2003). All these studies, however, were carried out with conventional molecular approaches, and analyzed a limited number of sequences, between 20 and 59 clones (DeLong et al. 1993; Rath et al. 1998; Acinas et al. 1999; Kellogg and Deming 2009). Moreover, several of the studies used fingerprinting techniques that could detect Operational Taxonomic Units (OTUs) but could not assign them to taxa (Ghiglione et al. 2009). Finally, at least in one case, no comparison of the PA bacteria with the FL bacteria was provided (Rath et al. 1998).

Previous studies could only analyze the most abundant taxa. These studies concluded that PA bacteria were less diverse than FL bacteria (Acinas et al. 1999; Hollibaugh et al. 2000; Ghiglione et al. 2007; Kellogg and Deming 2009). However, most of the diversity is in the less abundant taxa (Sogin et al. 2006). In 2006 Sogin et al. introduced the pyrosequencing approach, which provides hundreds of thousands of reads, increasing the number of OTUs by at least one order of magnitude, and allowing the detection of less abundant taxa. Although this approach still requires careful evaluation of possible biases and potential errors (Huse et al. 2007, 2010), its use has improved significantly the depth of knowledge about the structure of marine bacterial communities (Sogin et al. 2006; Huse et al. 2007, 2010; Pedrós-Alió 2012). In the present work, the richness and phylogenetic composition of FL and PA bacterial assemblages from the Northwest Mediterranean Sea were explored using pyrosequencing data. The high number of sequences provided by this technique allowed to consider the following subjects: (a) to compare the taxa richness of PA and FL assemblages; (b) to determine differences between PA and FL assemblages when the less abundant members are included and to determine the amount of taxa shared by the two assemblages; (c) to look for novel groups not described before within the PA bacterial pool; and (d) to compare PA assemblages from different marine areas.

## Materials and Methods

### Study area

**Figure 1:**
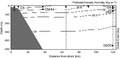
Density field along the transect sampled in the Northwest Mediterranean Sea between station A in the Blanes Bay Microbial Observatory (41º40′0.0″N, 2º48′0.0″E) and station D at the open sea (40º39′4.7″N, 2º51′1.6″E), showing the five sites sampled (filled squares) from the coast toward the open sea: station C at 5 m depth (C5), station CM at 44 m (CM44), and station D at 5 (D5), 65 (D65), and 500 m (D500). Filled circles show the stations sampled for pyrosequencing of the free-living (FL) fraction only.

A coast-to-offshore transect was sampled in the Northwest Mediterranean Sea during cruise MODIVUS between the 20th and the 23rd of September 2007 on board the RV “García del Cid”. The sampling started at a station close to the coast in the Blanes Bay Microbial Observatory (41º40′0.0″N, 2º48′0.0″E, station A in Fig. 1) and ended at an open sea station (station D) (40º39′4.7″N, 2º51′1.6″E) where samples were collected from the surface to 2000 m depth (the latter depth not shown in Fig. 1). For this study we considered samples only from 5 of the 10 stations sampled where samples for both fractions, FL and PA, were collected. Stations C5 and CM44 (Fig. 1, Table 1) were considered coastal stations and the remaining stations, D5, D65, and D500, were considered open sea stations.

**Table 1:** Summary of location and depth (m) of samples, total sequences before normalization (Tags), total operational taxonomic units (OTUs) at 0.03 level of clustering, percentage of singletons, Shannon diversity index (*H*′), richness (*S*), and evenness of the bacterial assemblages from the free-living (FL) and particle-attached (PA) samples

Sample	Latitude, longitude	Depth (m)	Tags	OTU (0.03)	Singletons% OTUs	*H*′	*S*	Evenness
FL_C5	41º39′5.6″N, 2º48′1.3″E	5	18405	1111	52.3	4.36	579	0.69
FL_CM44	41º24′5.9″N, 2º48′4.9″E	44	20926	386	39.4	3.89	482	0.63
FL_D5	40º39′4.7″N, 2º51′1.6″E	5	21937	367	41.2	3.82	354	0.65
FL_D65	40º39′4.7″N, 2º51′1.6″E	65	22744	521	42.1	4.54	616	0.71
FL_D500	40º39′4.7″N, 2º51′1.6″E	500	23336	816	45.6	4.25	732	0.64
PA_C5	41º39′5.6″N, 2º48′1.3″E	5	10299	1200	53.6	4.63	800	0.69
PA_CM44	41º24′5.9″N, 2º48′4.9″E	44	12814	1586	53.6	4.99	978	0.72
PA_D5	40º39′4.7″N, 2º51′1.6″E	5	13282	516	40.0	3.86	565	0.61
PA_D65	40º39′4.7″N, 2º51′1.6″E	65	18427	448	41.0	4.31	598	0.67
PA_D500	40º39′4.7″N, 2º51′28″E	500	12495	1328	33.4	4.84	810	0.72

Stations were named from the coast toward the open sea as follows: station C at 5 m depth (C5), station CM at 44 m depth (CM44), station D at 5 m depth (D5), station D at 65 m depth (D65), and station D at 500 m depth (D500). Station D65 was at the Deep Chlorophyll Maximum (DCM).

### Sampling

Samples were collected with Niskin bottles mounted on a rosette with a conductivity-temperature-depth profiler. Water was prefiltered through a 200-μm mesh and immediately processed on board. To collect microbial biomass, between 5 and 15 L of seawater was prefiltered through a 20-μm mesh and filtered through a 3 μm pore size Durapore filter (Millipore, Cork, Ireland) for the PA fraction (3–20 μm) and a 0.22-μm Sterivex filter (Durapore, Millipore) for the FL fraction (0.22–3 μm) in succession, using a peristaltic pump. The 3-μm filters were placed in vials which, as well as the 0.2-μm Sterivex units, were filled with 1.8 mL of lysis buffer (40 mmol l^−1^ EDTA, 50 mmol l^−1^ Tris-HCl, 0.75 mol l^−1^ sucrose) and stored at −80°C. DNA was extracted by a standard protocol using phenol/chloroform (details in Schauer et al. 2003). The same amount of DNA for every sample was sequenced.

### Sequencing and noise removal

The V6 region of the 16S rRNA was amplified with bacterial universal primers and the amplicons were pyrosequenced with a 454 Life Science GS-FLX sequencer at the Josephine Paul Bay Center, Marine Biological Laboratory in Woods Hole, Massachusetts, U.S.A. The protocols have been described in detail in Sogin et al. (2006), Huse et al. (2007), and on the Visualization and Analysis of Microbial Population Structures (VAMPS) webpage (http://vamps.mbl.edu/). For each read from the sequencer, the primer bases were trimmed from the beginning and the end, and low-quality sequences were removed in accordance with Huse et al. (2007). Sequences were flagged as low quality (1) when they were <50 nucleotides (Ns) in length, (2) when the start of the sequence did not exactly match a primer sequence, (3) when the sequences contained ambiguous Ns assignments with one or more unknown Ns, or (4) if the first five Ns of a tag sequence did not correspond to the expected run key (used to sort the pyrosequencing reads from different samples). Chimera checking and removal was performed using the Uchime option of USearch. This was done using a combination of reference set comparison (similar to Chimera Slayer) and de novo checking (directly comparing sequences within each PCR amplification). Taxonomy of V6 tags was assigned using the tag mapping methodology GAST (Global Alignment for Sequence Taxonomy) described in Sogin et al. (2006) and in the VAMPS webpage (http://vamps.mbl.edu/). This methodology uses reference databases of rRNA based on SILVA (Pruesse et al. 2007) and MUSCLE to align the tag sequences to the reference tags corresponding to the top 100 BLAST hits (Sogin et al. 2006). In addition, the tags were subjected to the preclustering method mentioned by Huse et al. (2010). All sequences obtained for this study are available at http://vamps.mbl.edu/ and have been deposited at NCBI Sequence Read Archive (SRA) under the accession number SRP001214.

### Tag clustering and normalization

The bacteria collected on the 3-μm filter were considered to be the PA fraction and the bacteria collected on the 0.2-μm Sterivex unit were the FL fraction. Normalization of sample size was achieved by randomly resampling the same number of reads for each sample based on the smallest sample size. This approach was carried out using the inbuilt command sub.sample in MOTHUR v1.22 (Schloss et al. 2009). The subsample dataset was then clustered in OTUs of decreasing genetic distance using MOTHUR v1.22 (Schloss et al. 2009) according to the furthest neighbor-clustering algorithm. To build the clusters in MOTHUR, we used the aligned version (April 2010) of SILVA (Pruesse et al. 2007). The output at each level for each distance level was then parsed to produce abundance tables of each OTU in each sample at different clustering levels. We focused our analysis on the 0.03 level of clustering. This level of clustering together with the noise removal approach (preclustering) are important to minimize the potential sequencing and/or polymerase chain reaction (PCR) errors that may have occurred during the amplification and pyrosequencing steps. For further analysis we grouped the OTUs into the lowest taxonomic level allowed through OTUs classification, this is in taxa, and in phyla.

### Analysis of sequences and comparison of assemblages

Rarefaction curves were calculated using Ecosim v 7.72 (Gotelli and Entsminger 2011). Nonmetric multidimensional scaling (NMDS) was carried out with a Bray–Curtis (BC) distance matrix using the “MASS” package (Venables and Ripley 2002) from R software (R Development Core Team 2012). Two different nonparametric multivariate analyses of the data, Permutational Multivariate Analysis of Variance (PERMANOVA) and Analysis Of Similarities (ANOSIM), were carried out using the “vegan” package (Oksanen et al. 2012) from R software (R Development Core Team 2012). ANOSIM and PERMANOVA were done using both BC distances matrices and Jaccard presence/absence similarity matrices and 9999 permutations. We used Metastats (White et al. 2009) for detection of significant differences between FL and PA assemblages in abundance of phyla and taxa.

### Calculation of richness, evenness, and diversity

Richness (*S*) was computed as the total number of OTUs (at the 0.03 level) in each sample. The Shannon index (*H*′) was used to determined diversity:

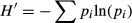
1
where *p*_i_ = *N*_*i*_/*N*, the number of individuals of species *i* divided by the total number of individuals in the sample (*N*). Finally, evenness was computed with the Pielou index:


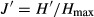
2
where *H*′ is the Shannon diversity index and *H*_max_ is the maximal possible Shannon diversity index if all the species were equally abundant:


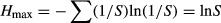
3
where *S* is the total number of OTUs (richness).

## Results

### OTUs richness

A total of 174,665 tags were obtained, 107,348 tags from FL samples and 67,317 from PA samples. We normalized the tag sequences obtained to the smallest sample size (10,299 tags) and we clustered them at 0.03 difference level obtaining 3588 OTUs. The average 293 singletons per sample in our dataset represented 44.2% of the total of the OTUs (Table 1). We grouped OTUs in taxa and phyla, obtaining 513 taxonomic groups and 30 phyla, for further analyses.

**Figure 2:**
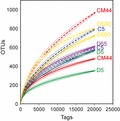
Rarefaction curves for free-living (continuous curves) and particle-attached (dashed curves) samples along the transect (sampled sites: C5, CM44, and D5) and at the vertical profile in station D (sampled sites: D5, D65, and D500). Difference level clusters of 0.03 were used. Curves for the same station are shown in the same color which corresponds to the color of the label. 95% confidence intervals are indicated by continuous curves for free-living samples and dashed curves for particle-attached samples, at both sides of the main curve. D65 curves cannot be differentiated in this graph and are shown with only one label.

Rarefaction curves were computed for the samples (Fig. 2). These curves allow comparisons of the OTU richness of each sample if the same number of tags had been obtained from all of them. The curves did not reach an asymptote. Rarefaction curves for the PA fraction reached higher number of OTUs per tags than the curves for the FL fraction in all samples except for sample D65 (Fig. 2). The 95% confidence intervals of the rarefaction curves for PA did not overlap with those of the curves for FL, except for the D65 sample (Fig. 2). Richness values (*S* in Table 1) were also higher in the PA fraction than in the FL fraction (*P* < 0.05, *t*-test for paired samples of *S* in Table 1) except again for sample D65. The D65 sample was at the Deep Chlorophyll Maximum (DCM) (Pommier et al. 2010). Evenness followed the same pattern as richness, but the small differences among samples (Table 1) were not significant (*P* = 0.24, *t*-test for paired samples). The Shannon diversity (*H*′) values (Table 1) calculated for the PA bacterial assemblages were slightly higher than those of corresponding FL assemblages but not significantly (*P* < 0.1, *t*-test for paired samples) (Table 1).

### Distribution of abundant and rare OTUs

**Figure 3:**
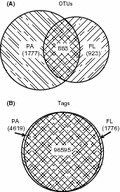
(A) Venn diagram showing operational taxonomic unit (OTU) distribution between fractions, and (B) Venn diagram showing tag distribution between fractions. The size of the circles is proportional to the number of OTUs in (A) and the number of tags in (B). Plots were obtained in http://bioinforx.com/free/bxarrays/venndiagram.

Among the 3588 OTUs found in this study, 25% appeared in samples from both fractions, 26% appeared only in FL samples, and 49% appeared only in PA samples (Fig. 3A). The distribution of the 102,990 tags from the 10 samples (obtained after normalization) between the two fractions showed that the shared OTUs were the most abundant ones: 94% tags appeared in both fractions, 4% tags only in the PA fraction, and 2% tags only in the FL fraction (Fig. 3B). OTUs exclusive to FL and PA bacterial assemblages, and therefore rare OTUs, represented only about 6% of total bacterial abundance.

### Distribution of bacterial assemblages

**Figure 4:**
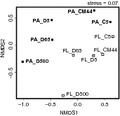
Nonmetric multidimensional scaling (NMDS) plot showing dissimilarities among the samples with Bray–Curtis distances. Samples are named according to their fraction (free living, [FL] and particle attached, [PA]), their location (stations C, CM, and D), and the depth where the water was collected (5, 44, 65, and 500 m). Open squares correspond to FL samples and filled squares correspond to PA samples. PA labels appeared in bold type.

The NMDS plot showed that FL and PA samples formed two separate groups (Fig. 4). There was also a separation of samples depending on their location (coastal or open sea) and there was a clear ordination of the samples with depth.

**Figure 5:**
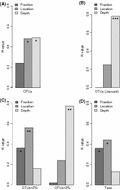
Barplots showing results of ANOSIM analysis (R value). (A) Analysis carried out with Bray–Cutis distance matrix, (B) analysis carried out with Jaccard similarity index matrix, (C) analysis carried out with Bray–Curtis distance matrices for abundant OTUs (>2% of total bacterial abundance in at least one of the samples) and rare OTUs (<2%), and (D) analysis carried with Bray–Curtis distance matrix for OTUs grouped in taxa (see M&M for criteria). The stars inside the bars indicate significance of ANOSIM values. Significance codes: ***(<0.001), **(<0.01), *(<0.05). The lack of stars indicates no signif-icance. ANOSIM, analysis of similarities; OTUs, operational taxonomic units.

In order to test whether these differences were significant we carried out ANOSIM tests with BC distance matrices. This nonparametric procedure tests whether distances within one group of samples and within other group of samples, for example, FL and PA samples, are shorter than distances between both groups. The R statistic for the differences between FL and PA samples had a value of 0.2 (black bar, Fig. 5A) that was not significant. On the other hand, ANOSIM showed significant differences between the coast and the open sea, and among depths (gray bars, Fig. 5A). We next carried out a PERMANOVA test, where the contributions of each factor could be calculated separately from the indirect effects of the other two. In this case, 19% of the variability was due to size fraction, whereas 29% and 30% of the variability was due to location and depth, respectively (these three values were highly significant). Thus, in the ANOSIM, the differences due to fraction were being masked by the larger variability due to the other two factors. In fact, looking at the NMDS diagram (Fig. 4) it is apparent that the three factors have to be important.

We repeated the analyses using presence/absence distance matrices (Jaccard similarity index). In this case fraction was not significant and it explained only 10% of the variability. The only differences were due to depth (Fig. 5B). This factor explained 32% of the variability. With presence/absence data, equal importance is given to abundant and rare OTUs. These results, considered together with the PERMANOVA results above, suggest that differences due to size fraction were caused by the abundant, and not by the rare OTUs. To check this point, we carried out analyses with BC distance matrices separately for the abundant (>2% of bacterial abundance in at least one of the samples) and rare (<2%) OTUs. In effect, size fraction was significant only for the abundant OTUs, whereas depth was more important for the rare ones (Fig. 5C). We did one last test grouping OTUs in taxa (see M&M for criteria). In this case, the effects of size fraction were significant (Fig. 5D) and the variability explained was 24%, close to that explained by location (30%) and higher than depth (23%). Depth was not significant according to ANOSIM.

In conclusion, the PA and FL assemblages were different, but these differences were not as large as those due to distance to shore and depth. The differences were due to the abundant OTUs and were particularly significant when OTUs were grouped into taxa.

### Taxonomic composition of the FL and PA bacterial assemblages: Phyla

**Figure 6:**
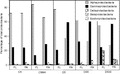
Percentage of *Proteobacteria* classes in the free-living (FL) and particle-attached (PA) samples at the five sampled sites (C5, CM44, D5, D65, and D500).

BC dissimilarity values calculated for the FL and PA samples grouped in phyla were higher at stations CM44, C5, and D500 than at stations D5 and D65 (Table S1 A). The *Proteobacteria* phylum was the main component of both bacterial assemblages (Table 2) representing on average ∼80% (±15%) of total bacteria. The contribution of *Proteobacteria* was higher in open sea than in coastal waters in both assemblages. The abundance of *Proteobacteria* was more similar between fractions in stations D5 and D65, whereas in stations C5, CM44, and D500 *Proteobacteria* were more abundant in the FL fraction (Table 2). Alpha*-Proteobacteria* and gamma-*Proteobacteria* were the most abundant *Proteobacteria* classes (Fig. 6). Alpha-*Proteobacteria* were more abundant in the FL fraction (55–84%) than in the PA fraction (28–71%) except for the coastal sample (C5) where they had similar values (Fig. 6, Table 2). On the contrary, gamma-*Proteobacteria* were more abundant in the PA fraction (24–60%) than in the FL fraction (13–33%) except, again, for the coastal sample (C5) where they had similar values (Fig. 6, Table 2). The high abundance of gamma-*Proteobacteria* in the PA fraction was especially noticeable at the three samples from the open sea location (D5, D65, and D500). In addition, the abundance of alpha*-Proteobacteria* was higher in the samples from the PA fraction at the stations closest to the coast (C5 and CM44) than in the water samples collected at the open sea (Fig. 6, Table 2B). Gamma-*Proteobacteria*, alpha-*Proteobacteria,* and epsilon-*Proteobacteria* were significantly different between FL and PA bacterial assemblages (*P* < 0.05 for the three classes, Metastast analysis) (Table S2 A). The significance of Gamma-*Proteobacteria* and alpha-*Proteobacteria* differences increased when the coastal station (C5) was removed from the analysis (*P* < 0.01, *P* < 0.05, respectively, Metastats analysis) (Table S2 B). Differences between alpha-*Proteobacteria* and gamma-*Proteobacteria* in FL and PA were lower in station C5. Removing *Proteobacteria* from the calculations increased the BC values (Table S1 B).

**Table 2:** Percentage of the most abundant phyla (≥1% of total bacterial abundance in at least one of the samples) and percentage of the main *Proteobacteria* classes, alpha- *Proteobacteria* (Alpha,) and gamma- *Proteobacteria* (Gamma) in (A) free-living (FL) and (B) particle-attached (PA) bacterial assemblages

	C5	CM4	D5	D65	D500
(A)					
FL samples					
*Actinobacteria*	0.4	0.7	0.1	1.4	1.1
*Bacteroidetes*	7.5	4.8	1.4	1.0	0.4
*Cyanobacteria*	15.7	0.8	2.7	4.9	0.2
*Deferribacteres*	2.1	1.3	0.7	1.2	3.2
*Firmicutes*	0.4	0.2	0.1	0.1	0.2
*Planctomycetes*	0.1	0	0	0.3	0.1
*Proteobacteria*	70.7	88.6	93.3	87.1	90.6
Alpha	71.7	84.0	77.0	61.7	55.1
Gamma	24.6	13.4	21.0	32.6	24.5
*Verrucomicrobia*	2.3	3.4	1.0	3.3	2.6
(B)					
PA samples					
*Actinobacteria*	0.7	1.0	0.5	1.2	4.7
*Bacteroidetes*	13.3	21.3	1.0	1.3	1.9
*Cyanobacteria*	17.2	6.1	0.9	5.5	1.0
*Deferribacteres*	1.2	1.0	0	0.5	2.7
*Firmicutes*	1.3	1.0	1.2	0.2	0.8
*Planctomycetes*	1.1	4.0	0.1	2.1	3.8
*Proteobacteria*	58.9	49.4	93.3	83.6	80.6
Alpha	71.6	65.8	37.5	33.2	27.9
Gamma	23.8	28.8	59.0	60.0	57.0
Verrucomicrobia	5.3	15.1	1.0	5.2	3.3

Stations referenced from the coast toward the open sea: station C at 5 m depth (C5), station CM at 44 m depth (CM44), station D at 5 m depth (D5), station D at 65 m depth (D65), and station D at 500 m depth (D500). The groups shown represent at least 1% of the total bacteria in at least one of the samples.

**Figure 7:**
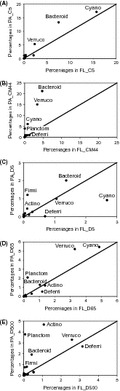
Relative enrichment of each abundant phyla (≥1% of total bacterial abundance in at least one of the samples), except *Proteobacteria,* in the free-living (FL) or particle-attached (PA) fractions at the five sampled sites: (A) C5, (B) CM44, (C) D5, (D) D65, and (E) D500. The solid line corresponds to the 1:1 line. *Bacteroidetes* (Bacteroid), C*yanobacteria* (Cyano), *Deferribacteres* (Deferri), *Planctomycetes* (Planctom), *Verrucomicrobia* (Verruco), *Firmicutes* (Firmi), and *Actinobacteria* (Actino).

Differences in abundance depending on the fraction were also detected within other less abundant phyla; we draw attention to the seven phyla representing at least 1% of the total abundance in at least one of the samples (Fig. 7, Table 2). *Cyanobacteria* represented >15% of the total bacterial abundance in the coastal sample (C5) for FL and PA fractions and, interestingly, this phylum was more abundant in PA than in FL samples except at the surface open sea station (D5). *Bacteroidetes* and *Verrucomicrobia* were always more abundant in the PA fraction, accounting for a sizable percentage of the total bacterial assemblage in the coastal stations (C5 and CM44) (Fig. 7A and B, Table 2B) where *Proteobacteria* were less abundant. Specifically, *Bacteroidetes* represented 13% and 21% in stations C5 and CM44, respectively. *Deferribacteres* were more abundant in the FL samples and *Actinobacteria* in the PA samples. *Planctomycetes* were well represented in the PA fraction except in the surface samples (C5 and D5). Contrarily, *Firmicutes* were more abundant in the surface samples in the PA fraction. The presence of *Planctomycetes* and *Firmicutes* in the FL fraction was negligible (Fig. 7A–E, Table 2A). Within the less abundant phyla only *Firmicutes* and *Planctomycetes* were significantly different between FL and PA bacterial assemblages (*P* < 0.05, Metastats analysis) (Table S3).

### Taxonomic composition of FL and PA bacterial assemblages: Taxa

**Figure 8:**
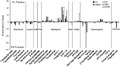
Enrichment of abundant bacterial taxa (≥2% of total bacterial abundance in at least one of the samples) in the free-living (FL) or the particle-attached (PA) fractions at the five sampled sites (C5, CM44, D5, D65, and D500). Enrichment was calculated by subtracting the percentage of every genus in PA from the percentage of every genus in FL. The positive values denote enrichment in the FL fraction and negative values denote enrichment in the PA fraction. Vertical lines separate groups of different phyla or classes. *Bacteroidetes* (Bacteroid), *Cyanobacteria* (Cyano), *Deferribacteres* (Def), *Planctomycetes* (Plan), alpha-*Proteobacteria* (Alphaprot), beta-*Proteobacteria* (Beta), delta*-Proteobacteria* (Delta), gamma-*Proteobacteria* (Gammaprot), and *Verrucomicrobia* (Verruco).

“Pelagibacter” (alpha-*Proteobacteria*) and *Alteromonas* (gamma*-Proteobacteria*) were the two most abundant genera in most samples and presented different contributions to the FL and PA fractions. “Pelagibacter” was much more abundant in FL samples, whereas *Alteromonas* was more abundant in PA samples, mainly in the open sea location (Fig. 8). In general, as already mentioned, alpha*-Proteobacteria* genera were more abundant in FL samples, whereas gamma-*Proteobacteria* genera were more abundant in PA, the only exception being an unknown genus from the family SAR86 from the order *Oceanospirillales* that was more abundant in FL samples (Fig. 8). In accordance with the phyla distribution already described, the taxa belonging to *Cyanobacteria* (*Synechococcus* and *Prochlorococcus*), *Bacteroidetes, Verrucomicrobia*, and *Planctomycetes*, although found in low abundance, were more abundant in PA than in FL samples. Also found in low abundance, SAR324 and *Nitrospina* (delta-*Proteobacteria*) were mainly present in the FL deepest open sea sample and *Ralstonia* (beta*-Proteobacteria*) were more abundant in the PA deepest open sea sample than in the FL one (Fig. 8). No genera from the *Firmicutes* and *Actinobacteria* phyla were abundant enough (≥2%) to fulfill the criterion to appear in this list. “Pelagibacter”, SAR86, and *Planctomycetaceae* were the only taxa significantly different between FL and PA assemblages (Table S4 A). However, removing from the analysis the coastal stations (C5, CM44) where *Alteromonas* were equally abundant (*P* = 0.4, *t*-test for paired samples), the taxa significantly different between FL and PA assemblages were SAR86, SAR11, *Alteromonas*, “Pelagibacter”, and *Rhodospirillaceae* (*P* < 0.05, Metastats Analysis) (Table S4 B).

## Discussion

The rarefaction curves and the richness values clearly showed that PA bacterial assemblages were richer than the FL bacterial assemblages (Fig. 2A, Table 1). Although this has been found in a study of the bacterial community in a coastal lagoon (LaMontagne and Holden 2003), most studies in the Mediterranean waters (Acinas et al. 1999; Ghiglione et al. 2007) and in other marine ecosystems (Hollibaugh et al. 2000; Kellogg and Deming 2009) reported higher richness of the FL bacteria. None of these studies, including ours, reached saturation of the collectors curves (Fig. 2) and one might argue that comparisons of richness values among samples were unwarranted. However, the slopes of the curves for PA bacteria were clearly steeper than those for FL bacteria and the 95% confidence intervals of the curves for PA did not overlap with those of the curves for FL (Fig. 2). Therefore, the conclusion that PA samples were richer than FL samples is robust.

The differences with previous studies might be due to the different fractionation schemes used in each study. LaMontagne and Holden (2003) also used a 3-μm filter to collect the PA fraction, but the filters in the other three studies had smaller pore sizes (0.8, 1, and 1.2 μm). However, the latter fractionation should have resulted in higher richness in the PA fraction. More likely, the higher richness of PA samples in this study was due to the use of different techniques for DNA sequencing providing different number of sequences. Previous studies used conventional molecular techniques, which retrieved only a few clones (between 18 and 45) and OTUs (between 4 and 60) (Acinas et al. 1999; Hollibaugh et al. 2000; Kellogg and Deming 2009). The number of OTUs in the present work was two orders of magnitude higher than in those previous studies. Also, the number of OTUs exclusive to each fraction was higher in the PA fraction than in the FL fraction (Fig. 3A). As far as we know, a higher number of OTUs in the PA fraction than in the FL fraction has been never reported before. The co-occurrence of several OTUs in both fractions, 25%, has been already described for the Mediterranean Sea (between 24% and 69%, Moeseneder et al. 2001; Ghiglione et al. 2009). The pyrosequencing approach we used here also allowed the detection of rare taxa (Sogin et al. 2006; Pedrós-Alió 2012) not seen before with other techniques revealing the larger richness of PA bacterial assemblages that was hidden in previous studies (Acinas et al. 1999; Hollibaugh et al. 2000; Ghiglione et al. 2007).

The taxonomic composition of phytoplankton communities necessarily influences that of bacterial assemblages and provides several ecological niches where specialized bacteria can live (i.e., Teeling et al. 2012). Therefore, a higher complexity of the phytoplankton community at the DCM (D65) (Estrada and Salat 1989; Ghiglione et al. 2007) is probably responsible for the similar richness values found between the FL and PA bacterial assemblages in that sample (Fig. 2, Table 1). Higher number and quality of particles at the coastal (C5, CM44) and the deep samples (D500) than at the open sea surface (D5) could explain the higher richness of the PA fraction as well as the higher richness of the whole bacterial assemblage at those sites (Fig. 2, Table 1).

Comparisons among samples showed that even though there were significant differences between PA and FL assemblages, there were larger differences in community composition due to sample location and depth. It was also shown that the differences between PA and FL were due to the more abundant OTUs. These differences were particularly significant when OTUs were grouped in taxa.

DeLong et al. (1993), analyzing a very limited number of sequences (20 clones for FL and 18 clones for PA from one single sample in a coastal location), found the majority of FL clones to belong to alpha-*Proteobacteria* and the majority of PA clones to belong to *Cytophaga* (now *Bacteroidetes*), *Planctomycetes*, and gamma-*Proteobacteria*. This distribution of groups agrees fairly well with what we found analyzing three orders of magnitude more sequences: alpha*-Proteobacteria* dominated the FL assemblage and gamma-*Proteobacteria,* and less abundant groups (*Firmicutes* and *Planctomycetes*) dominated the PA fraction (Figs. 6, 7; Table 2). We have not found significant differences in *Bacteroidetes* between FL and PA bacterial assemblages (Table S3), probably due to masking of significance by the high abundance of this phylum in the PA fraction at coastal waters (Table 2). This high abundance, however, revealed the importance of *Bacteroidetes* in the PA fraction, at least at the coastal station as was also found by DeLong et al. (1993). The low abundance of *Actinobacteria* and *Verrucomicrobia* may have also masked any significance of their higher abundance in the PA bacteria than in the FL bacteria.

The *Proteobacteria* phylum was important in explaining differences between FL and PA bacterial assemblages, but once *Proteobacteria* were removed from the analysis, the less abundant groups were also important. The present data corroborate that FL and PA bacteria are phylogenetically different (DeLong et al. 1993; Acinas et al. 1999; LaMontagne and Holden 2003). However, the higher number of sequences has added new phyla (*Actinobacteria, Firmicutes*, and *Verrucomicrobia*) to the PA bacteria pool (Rath et al. 1998) and has shown that the PA bacterial assemblages are different from each other at different locations and depths.

The presence of cyanobacterial clones in the PA bacteria was shown by DeLong et al. (1993) in their study on the Santa Barbara channel. These authors attributed such clones to chloroplast rRNA sequences. In the present work, chloroplast sequences were removed from the analyses and the high abundance of *Cyanobacteria* in the PA fraction was presumably due to their retention on the 3-μm filter during filtration (LaMontagne and Holden 2003; Ghiglione et al. 2007).

It is well known that *Alteromonas* has higher nutrient requirements than “Pelagibacter”*,* which thrive in oligotrophic conditions (Schattenhofer et al. 2009). Thus, it seems likely that *Alteromonas* and other gamma-*Proteobacteria* attach to particles to avoid the nutrient-depleted conditions in the surrounding waters, whereas “Pelagibacter” can remain living free in the water column (Fig. 8). This distribution has been already described for the Northwest Mediterranean Sea (Acinas et al. 1999). DeLong et al. (1993) attributed the high abundance of gamma-*Proteobacteria* groups in the PA bacteria to the ability of these groups to bind to and degrade high molecular weight compounds (Karner and Herndl 1992; Pinhassi et al. 1999; Bauer et al. 2006). However, it has been shown that *Oceanospirillum* sp., in the order *Oceanospirillales* (gamma-*Proteobacteria*), grows much better in low-nutrient synthetic medium than other gamma-*Proteobacteria* (Pernthaler et al. 2001). This peculiarity could explain the enrichment of SAR86 (*Oceanospirillales*) in the FL fraction (Fig. 8). Less abundant taxa (SAR324 and *Ralstonia*) have been also found differentially distributed, but the differences were not significant probably due to their low abundance (Fig. 8). Clearly, the large number of sequences obtained by pyrosequencing has allowed a higher taxonomic resolution of FL and PA bacteria taxonomic composition than other conventional methods would allow (DeLong et al. 1993; Rath et al. 1998; Acinas et al. 1999).

The phyla and taxa enrichment in FL and PA bacterial assemblages reflects that some bacteria are specialized in the transformation of particulate organic matter and live mainly attached to particles (Pedrós-Alió and Brock 1983; DeLong et al. 1993). However, PA bacteria must be capable of surviving freely in the water column so they can colonize new particles (Pedrós-Alió and Brock 1983; Ghiglione et al. 2007). This may explain the higher similarities between FL and PA bacterial assemblages at the coastal station (C5) (Figs. 6, 7, 8), where a high number of particles in the water column due to land–sea interaction would contribute to increase the presence of PA bacteria living free in the water waiting to colonize new particles. On the other hand, the differences in phyla distribution within the PA samples point to an effect of the organic matter compounds of the particles on the bacterial composition (Pinhassi et al. 1999), as presumably, the organic composition of the particles would be different depending on the location of the samples.

## Supporting Information

Table S1 Bray–Curtis dissimilarity values between Free-Living (FL) and Particle-Attached (PA) samples grouped in phyla. (A) all phyla, and (B) all phyla except *Proteobacteria*. Stations were named from the coast toward the open sea as follows: station C at 5 m depth (C5), station CM at 44 m depth (CM44), station D at 5 m depth (D5), station D at 65 m depth (D65), and station D at 500 m depth (D500). BC is a statistic used to quantify the compositional dissimilarity between two different sites based on counts at each site that takes values between 0 and 1 with 0 indicating total similarity and 1 indicating total dissimilarity.

Table S2 Metastast analysis testing the significant contribution of *Proteobacteria* classes to the differences between Free-Living (FL) and Particle-Attached (PA) bacterial assemblages. (A) Analysis with all the samples and (B) analysis with all the samples except the coastal station (C5). Gamma-*Proteobacteria* (Gamma), Alpha-Proteobacteria (Alpha), Epsilon-*Proteobacteria* (Epsilon), Beta-*Proteobacteria* (Beta), and Delta-*Proteobacteria* (Delta). Significant *P*-values are indicated in bold type.

Table S3 Metastast analysis testing the significant contributions of the most abundant phyla (≥1% of total bacterial abundance in at least one of the samples) to the differences between Free-Living (FL) and Particle-Attached (PA) bacterial assemblages. Significant *P*-values are indicated in bold type.

Table S4 Metastast analysis testing the significant contribution of the most abundant taxa (≥2% of total bacterial abundance in at least one of the samples) to the differences between Free-Living (FL) and Particle- Attached (PA) bacterial assemblages. (A) Analysis with all the samples, and (B) analysis with all the samples except the coastal station (C5). Significant *P*-values are indicated in bold type.
