# Supplementary material for: Taxonomic composition of the particle-attached and free-living bacterial assemblages in the Northwest Mediterranean Sea analyzed by pyrosequencing of the 16S rRNA
Source: Microbiologyopen. 2013 May 31;2(4):541–52. doi: 10.1002/mbo3.92 (PMC3948605; doi:10.1002/mbo3.92)
Supplement: Table S1 — Bray–Curtis dissimilarity values between Free-Living (FL) and Particle-Attached (PA) samples grouped in phyla. (A) all phyla, and (B) all phyla except Proteobacteria. Stations were named from the coast toward the open sea as follows: station C at 5 m depth (C5), station CM at 44 m depth (CM44), station D at 5 m depth (D5), station D at 65 m depth (D65), and station D at 500 m depth (D500). BC is a statistic used to quantify the compositional dissimilarity between two different sites based on counts at each site that takes values between 0 and 1 with 0 indicating total similarity and 1 indicating total dissimilarity. [file mbo30002-0541-sd1.doc]

Table S1. Bray-Curtis dissimilarity values between Free-Living (FL) and Particle Attached (PA) samples grouped in phyla. A) all phyla and B) all phyla except *Proteobacteria.* Stations were named from the coast towards the open sea as follows: Station C at 5 m depth (C5), station CM at 44 m depth (CM44), station D at 5 m depth (D5), station D at 65 m depth (D65) and station D at 500 m depth (D500). BC is a statistic used to quantify the compositional dissimilarity between two different sites based on counts at each site that takes values between 0 and 1 with 0 indicating total similarity and 1 indicating total dissimilarity,

A)

|  | Fl_C5 | Fl_CM44 | FL_D5 | Fl_D65 | Fl_D500 | PA_C5 | PA_CM44 | PA_D5 | PA_D65 |
| --- | --- | --- | --- | --- | --- | --- | --- | --- | --- |
|  |  |  |  |  |  |  |  |  |  |
| Fl_CM44 | 0.19 |  |  |  |  |  |  |  |  |
| FL_D5 | 0.23 | 0.07 |  |  |  |  |  |  |  |
| Fl_D65 | 0.19 | 0.06 | 0.07 |  |  |  |  |  |  |
| Fl_D500 | 0.23 | 0.06 | 0.07 | 0.07 |  |  |  |  |  |
| PA_C5 | 0.13 | 0.30 | 0.35 | 0.29 | 0.36 |  |  |  |  |
| PA_CM44 | 0.32 | 0.40 | 0.44 | 0.39 | 0.45 | 0.22 |  |  |  |
| PA_D5 | 0.24 | 0.07 | 0.03 | 0.09 | 0.07 | 0.35 | 0.45 |  |  |
| PA_D65 | 0.19 | 0.09 | 0.10 | 0.05 | 0.11 | 0.26 | 0.35 | 0.12 |  |
| PA_D500 | 0.22 | 0.12 | 0.16 | 0.12 | 0.11 | 0.32 | 0.38 | 0.14 | 0.11 |

B)

|  | Fl_C5 | Fl_CM44 | FL_D5 | Fl_D65 | Fl_D500 | PA_C5 | PA_CM44 | PA_D5 | PA_D65 |
| --- | --- | --- | --- | --- | --- | --- | --- | --- | --- |
|  |  |  |  |  |  |  |  |  |  |
| Fl_CM44 | 0.51 |  |  |  |  |  |  |  |  |
| FL_D5 | 0.64 | 0.52 |  |  |  |  |  |  |  |
| Fl_D65 | 0.51 | 0.40 | 0.39 |  |  |  |  |  |  |
| Fl_D500 | 0.70 | 0.47 | 0.65 | 0.47 |  |  |  |  |  |
| PA_C5 | 0.20 | 0.57 | 0.72 | 0.57 | 0.78 |  |  |  |  |
| PA_CM44 | 0.55 | 0.64 | 0.77 | 0.63 | 0.81 | 0.37 |  |  |  |
| PA_D5 | 0.71 | 0.48 | 0.39 | 0.60 | 0.65 | 0.74 | 0.79 |  |  |
| PA_D65 | 0.54 | 0.49 | 0.48 | 0.20 | 0.60 | 0.49 | 0.52 | 0.64 |  |
| PA_D500 | 0.69 | 0.50 | 0.71 | 0.53 | 0.43 | 0.69 | 0.64 | 0.61 | 0.51 |
