# Supplementary material for: Taxonomic composition of the particle-attached and free-living bacterial assemblages in the Northwest Mediterranean Sea analyzed by pyrosequencing of the 16S rRNA
Source: Microbiologyopen. 2013 May 31;2(4):541–52. doi: 10.1002/mbo3.92 (PMC3948605; doi:10.1002/mbo3.92)
Supplement: Table S2 — Metastast analysis testing the significant contribution of Proteobacteria classes to the differences between Free-Living (FL) and Particle-Attached (PA) bacterial assemblages. (A) Analysis with all the samples and (B) analysis with all the samples except the coastal station (C5). Gamma-Proteobacteria (Gamma), Alpha-Proteobacteria (Alpha), Epsilon-Proteobacteria (Epsilon), Beta-Proteobacteria (Beta), and Delta-Proteobacteria (Delta). Significant P-values are indicated in bold type. [file mbo30002-0541-sd2.doc]

Table S2. Metastast analysis testing the significant contribution of *Proteobacteria* classes to the differences between Free-Living (FL) and Particle Attached (PA) bacterial assemblages. A) Analysis with all the samples and B) analysis with all the samples except the coastal station (C5). Gamma-*Proteobacteria* (Gamma), Alpha-*Proteobacteria* (Alpha), Epsilon-*Proteobacteria* (Epsilon), Beta-*Proteobacteria* (Beta), Delta-*Proteobacteria* (Delta). Significant p-values are indicated in bold type.

A)

|  | FL |  | PA |  |  |
| --- | --- | --- | --- | --- | --- |
|  | Mean  abundance (%) | std.error | Mean  abundante (%) | std.error | p-value |
|  |  |  |  |  |  |
| Gamma | 0.23 | 0.03 | 0.46 | 0.08 | **0.02** |
| Alpha | 0.70 | 0.05 | 0.47 | 0.09 | **0.04** |
| Epsilon | 0.0004 | 0.00 | 0.002 | 0.00 | **0.05** |
| Beta | 0.01 | 0.00 | 0.02 | 0.01 | 0.15 |
| Delta | 0.06 | 0.03 | 0.05 | 0.01 | 0.67 |

B)

|  | FL |  | PA |  |  |
| --- | --- | --- | --- | --- | --- |
|  | Mean  abundance (%) | std.error | Mean  abundance (%) | std.error | p-value |
|  |  |  |  |  |  |
| Gamma | 22.88 | 0.04 | 51.21 | 0.07 | **0.01** |
| Alpha | 69.42 | 0.07 | 41.07 | 0.08 | **0.02** |
| Epsilon | 0.04 | 0.00 | 0.17 | 0.00 | 0.09 |
| Beta | 0.35 | 0.00 | 2.41 | 0.01 | 0.15 |
| Delta | 7.32 | 0.04 | 5.13 | 0.01 | 0.62 |
