# Supplementary material for: Taxonomic composition of the particle-attached and free-living bacterial assemblages in the Northwest Mediterranean Sea analyzed by pyrosequencing of the 16S rRNA
Source: Microbiologyopen. 2013 May 31;2(4):541–52. doi: 10.1002/mbo3.92 (PMC3948605; doi:10.1002/mbo3.92)
Supplement: Table S3 — Metastast analysis testing the significant contributions of the most abundant phyla (≥1% of total bacterial abundance in at least one of the samples) to the differences between Free-Living (FL) and Particle-Attached (PA) bacterial assemblages. Significant P-values are indicated in bold type. [file mbo30002-0541-sd3.doc]

Table S3. Metastast analysis testingthe significant contributions of the most abundant phyla (≥ 1% of total bacterial abundance in at least one of the samples) to the differences between Free-Living (FL) and Particle Attached (PA) bacterial assemblages. Significant p-values are indicated in bold type.

|  | FL |  | PA |  |  |
| --- | --- | --- | --- | --- | --- |
|  | Mean  abundance (%) | std.error | Mean  abundance (%) | std.error | p-value |
|  |  |  |  |  |  |
| *Firmicutes* | 0.00 | 0.00 | 0.01 | 0.00 | **0.00** |
| *Planctomycetes* | 0.00 | 0.00 | 0.02 | 0.01 | **0.01** |
| *Verrrucomicrobia* | 0.03 | 0.00 | 0.06 | 0.02 | 0.17 |
| *Proteobacteria* | 0.87 | 0.04 | 0.74 | 0.08 | 0.17 |
| *Bacteroidetes* | 0.03 | 0.01 | 0.08 | 0.04 | 0.26 |
| *Actinobacteria* | 0.01 | 0.00 | 0.02 | 0.01 | 0.30 |
| *Deferribacteres* | 0.02 | 0.00 | 0.01 | 0.00 | 0.34 |
| *Cyanobacteria* | 0.05 | 0.03 | 0.06 | 0.03 | 0.71 |
