# Supplementary material for: Taxonomic composition of the particle-attached and free-living bacterial assemblages in the Northwest Mediterranean Sea analyzed by pyrosequencing of the 16S rRNA
Source: Microbiologyopen. 2013 May 31;2(4):541–52. doi: 10.1002/mbo3.92 (PMC3948605; doi:10.1002/mbo3.92)
Supplement: Table S4 — Metastast analysis testing the significant contribution of the most abundant taxa (≥2% of total bacterial abundance in at least one of the samples) to the differences between Free-Living (FL) and Particle- Attached (PA) bacterial assemblages. (A) Analysis with all the samples, and (B) analysis with all the samples except the coastal station (C5). Significant P-values are indicated in bold type. [file mbo30002-0541-sd4.doc]

Table S4. Metastast analysis testing the significant contribution of the most abundant taxa (≥ 2% of total bacterial abundance in at least one of the samples) to the differences between Free-Living (FL) and Particle Attached (PA) bacterial assemblages. A) Analysis with all the samples and B) analysis with all the samples except the coastal station (C5). Significant p-values are indicated in bold type.

A)

|  | FL |  | PA |  |  |
| --- | --- | --- | --- | --- | --- |
|  | Mean  abundance (%) | std.error | Mean  abundance (%) | std.error | p-value |
| "Pelagibacter" | 0.30 | 0.05 | 0.15 | 0.02 | **0.01** |
| SAR86 | 0.06 | 0.01 | 0.03 | 0.01 | **0.03** |
| *Planctomycetaceae* | 0.00 | 0.00 | 0.02 | 0.01 | **0.05** |
| *Alteromonas* | 0.05 | 0.02 | 0.23 | 0.09 | 0.06 |
| SAR116 | 0.09 | 0.03 | 0.03 | 0.01 | 0.07 |
| Alpha-*Proteobacteria* | 0.02 | 0.00 | 0.01 | 0.00 | 0.09 |
| SAR11 | 0.08 | 0.01 | 0.06 | 0.01 | 0.09 |
| *Flavobacteriales* | 0.00 | 0.00 | 0.01 | 0.01 | 0.10 |
| *Rhodospirillaceae* | 0.04 | 0.01 | 0.03 | 0.00 | 0.11 |
| *Vibrionaceae* | 0.00 | 0.00 | 0.01 | 0.01 | 0.16 |
| *Roseibacillus* | 0.00 | 0.00 | 0.03 | 0.02 | 0.18 |
| *Vibrio* | 0.01 | 0.00 | 0.02 | 0.01 | 0.18 |
| SAR324 | 0.04 | 0.03 | 0.01 | 0.00 | 0.18 |
| *Rhodobacteraceae* | 0.12 | 0.03 | 0.07 | 0.02 | 0.25 |
| *Ralstonia* | 0.00 | 0.00 | 0.01 | 0.01 | 0.26 |
| *Owenweeksia* | 0.00 | 0.00 | 0.01 | 0.01 | 0.27 |
| Gamma-*Proteobacteria* | 0.03 | 0.01 | 0.05 | 0.02 | 0.31 |
| *Fluviicola* | 0.00 | 0.00 | 0.01 | 0.01 | 0.37 |
| *Enterobacteriaceae* | 0.01 | 0.01 | 0.02 | 0.01 | 0.37 |
| *Coraliomargarita* | 0.00 | 0.00 | 0.01 | 0.01 | 0.40 |
| *Flavobacteriaceae* | 0.02 | 0.01 | 0.04 | 0.02 | 0.45 |
| SAR406 | 0.02 | 0.00 | 0.01 | 0.01 | 0.63 |
| *Synechococcus* | 0.03 | 0.02 | 0.05 | 0.03 | 0.64 |
| *Nitrospina* | 0.01 | 0.01 | 0.00 | 0.00 | 0.64 |
| *Rickettsiales* | 0.01 | 0.01 | 0.01 | 0.01 | 0.83 |
| *Oceanobacter* | 0.03 | 0.02 | 0.02 | 0.01 | 0.87 |
| *Prochlorococcus* | 0.02 | 0.01 | 0.02 | 0.01 | 0.92 |
| *Haliea* | 0.01 | 0.00 | 0.01 | 0.01 | 0.92 |

B)

|  | FL |  | PA |  |  |
| --- | --- | --- | --- | --- | --- |
|  | Mean  abundance (%) | std.error | Mean  abundance (%) | std.error | p-value |
| SAR86 | 0.05 | 0.01 | 0.01 | 0.00 | **0.00** |
| *Alteromonas* | 0.06 | 0.01 | 0.36 | 0.08 | **0.01** |
| SAR11 | 0.08 | 0.01 | 0.04 | 0.01 | **0.01** |
| "Pelagibacter" | 0.30 | 0.04 | 0.13 | 0.02 | **0.01** |
| *Rhodospirillaceae* | 0.04 | 0.01 | 0.02 | 0.00 | **0.04** |
| *Vibrionaceae* | 0.00 | 0.00 | 0.02 | 0.01 | 0.08 |
| *Haliea* | 0.01 | 0.00 | 0.00 | 0.00 | 0.11 |
| SAR116 | 0.08 | 0.04 | 0.02 | 0.01 | 0.12 |
| *Vibrio* | 0.01 | 0.00 | 0.04 | 0.02 | 0.13 |
| *Enterobacteriaceae* | 0.01 | 0.01 | 0.03 | 0.01 | 0.15 |
| Gamma-*Proteobacteria* | 0.03 | 0.01 | 0.08 | 0.03 | 0.16 |
| SAR324 | 0.04 | 0.03 | 0.01 | 0.00 | 0.22 |
| Alpha-*Proteobacteria* | 0.02 | 0.00 | 0.01 | 0.00 | 0.24 |
| *Ralstonia* | 0.00 | 0.00 | 0.02 | 0.02 | 0.27 |
| *Flavobacetriaceae* | 0.02 | 0.01 | 0.00 | 0.00 | 0.29 |
| *Fluviicola* | 0.01 | 0.01 | 0.00 | 0.00 | 0.35 |
| *Planctomycetaceae* | 0.00 | 0.00 | 0.02 | 0.01 | 0.42 |
| *Owenweeksia* | 0.01 | 0.01 | 0.00 | 0.00 | 0.47 |
| *Roseibacillus* | 0.02 | 0.02 | 0.01 | 0.00 | 0.59 |
| *Flavobacteriales* | 0.01 | 0.01 | 0.00 | 0.00 | 0.60 |
| *Prochlorococcus* | 0.02 | 0.01 | 0.01 | 0.01 | 0.64 |
| *Oceanobacter* | 0.03 | 0.02 | 0.04 | 0.02 | 0.69 |
| *Rhodobacteraceae* | 0.11 | 0.03 | 0.09 | 0.04 | 0.77 |
| *Rickettsiales* | 0.00 | 0.00 | 0.00 | 0.00 | 0.81 |
| *Nitrospina* | 0.01 | 0.01 | 0.01 | 0.00 | 0.86 |
| *Coraliomargarita* | 0.00 | 0.00 | 0.00 | 0.00 | 0.88 |
| SAR406 | 0.02 | 0.00 | 0.01 | 0.01 | 0.90 |
| *Synechococcus* | 0.01 | 0.01 | 0.01 | 0.01 | 0.95 |
